# Cytological, genetic and transcriptomic characterization of a cucumber albino mutant

**DOI:** 10.3389/fpls.2022.1047090

**Published:** 2022-10-20

**Authors:** Jinqiang Yan, Bin Liu, Zhenqiang Cao, Lin Chen, Zhaojun Liang, Min Wang, Wenrui Liu, Yu'e Lin, Biao Jiang

**Affiliations:** ^1^ Vegetable Research Institute, Guangdong Academy of Agricultural Sciences, Guangzhou, China; ^2^ Guangdong Key Laboratory for New Technology Research of Vegetables, Guangdong Academy of Agricultural Sciences, Guangzhou, China; ^3^ Hami-melon Research Center, Xinjiang Academy of Agricultural Sciences, Urumqi, China

**Keywords:** albino mutant, cucumber, recessive, chloroplast deficiency, transcriptome

## Abstract

Photosynthesis, a fundamental process for plant growth and development, is dependent on chloroplast formation and chlorophyll synthesis. Severe disruption of chloroplast structure results in albinism of higher plants. In the present study, we report a cucumber albino *alc* mutant that presented white cotyledons under normal light conditions and was unable to produce first true leaf. Meanwhile, *alc* mutant could grow creamy green cotyledons under dim light conditions but died after exposure to normal light irradiation. No chlorophyll and carotenoid were detected in the *alc* mutant grown under normal light conditions. Using transmission electron microscopy, impaired chloroplasts were observed in this mutant. The genetic analysis indicated that the albino phenotype was recessively controlled by a single locus. Comparative transcriptomic analysis between the *alc* mutant and wild type revealed that genes involved in chlorophyll metabolism and the methylerythritol 4-phosphate pathway were affected in the *alc* mutant. In addition, three genes involved in chloroplast development, including two *FtsH* genes and one *PPR* gene, were found to have negligible expression in this mutant. The quality of RNA sequencing results was further confirmed by real-time quantitative PCR analysis. We also examined 12 homologous genes from *alc* mutant in other plant species, but no genetic variation in the coding sequences of these genes was found between *alc* mutant and wild type. Taken together, we characterized a cucumber albino mutant with albinism phenotype caused by chloroplast development deficiency and this mutant can pave way for future studies on plastid development.

## Introduction

Chloroplasts, which are DNA-containing organelles play crucial roles in attuning plant development and plant interaction with environmental cues. At the time of illumination, the chloroplast develops from proplastids *via* the process of photomorphogenesis (Pogson et al., 2015; Chan et al., 2016). The chloroplast is the site of photosynthesis and production of hormones (e.g., abscisic acid, jasmonic acid, and salicylic acid, and other major metabolites (Unlu et al., 2014). Its abnormal development or accumulation of pigments inside itself could affect photosynthesis and further disrupt plant growth and biomass yield (Wang et al., 2016; Shi et al., 2017; Xiong et al., 2017; Gotoh et al., 2018).

Leaf color mutation has been widely reported in many plant species. In most of the chlorophyll-less mutants and chlorophyll-deficient mutants, a sudden increase in the production of reactive oxygen species (ROS) was detected after exposure to light conditions (Sakowska et al., 2018; Li et al., 2019). The ROS accumulation induced by excessive light could effectuate oxidative damage in plants, resulting in leaf bleaching or leading to plant death. Natural or induced albino mutants were frequently identified and characterized among different kinds of leaf color mutations, especially in *Arabidopsis* and rice. For example, some formation of albino mutants was affected by environmental factors and conditionally green-revertible. Disruption of *OsABCI8* resulted in the development of albino leaves in rice under continuous rainy days; nevertheless, the leaves gradually turned green following rainy days (Zeng et al., 2017). Mutations in the gene *OsTCD5* encoding a monooxygenase, or *OsTCD11* encoding the ribosomal small subunit protein S6 in chloroplasts (RPS6) resulted in a temperature-sensitive albino mutant; the leaves displayed albinism at low temperatures but turned green at high temperatures (Wang et al., 2016). Mutation in gene *FLN2* encoding fructokinase-like protein2 shows opposite phenotype; the *fln2* mutant is albino at high temperatures (Qiu et al., 2018). There are other mutants that only exhibit albino phenotype at certain development stages. Disruption of a pentatricopeptide repeat protein causes an albino phenotype during the seedling stage but the plants are able to turn green during plant growth and development (Su et al., 2012). A mutation in SEEDLING PLASTID DEVELOPMENT1 resulted in albino cotyledons, but these plants appeared similar to the wild type plants once the initial true leaves developed and the seedlings were transferred to the soil (Ruppel et al., 2011).

Somatic albino mutants were also described with expression of genes involved in chlorophyll biosynthesis and chloroplast development affected in mutated leaves (Ma et al., 2018; Lu et al., 2019). Some lethal mutations are caused by deficiency in chloroplast development. Loss of function of *DXS1* leads to an albino phenotype in tomato with premature lethality performance (Garcia-Alcazar et al., 2017). A single-nucleotide mutation in the plastid ribosomal protein produces abnormal chloroplasts and causes seedling lethality in rice (Zhao et al., 2016).

Cucumber (*Cucumis sativus* L.), belonging to the Cucurbitacea family is an important vegetable crop worldwide. Cucumber albino mutation was only reported by Iida and Amano in 1991 (Iida and Amano, 1991), which was induced by irradiation. However, no further studies have been carried out since then. In the present study, we reported a spontaneous mutation of an albino mutant from the cucumber inbred line “g32”, which exhibited white cotyledons and hypocotyl, and died before developing any true leaves. We aim to decipher the potential mechanism of albinism formation in this cucumber albino mutant *via* cytologic, genetic and transcriptomica characterization.

## Materials and methods

### Plant materials

Cucumber inbred line g32 was used in this study. The inbred line was provided by Vegetable Research Institute, Guangdong Academy of Agricultural Sciences Guangzhou, China. Seeds from a self-pollinated g32 cucumber fruit were soaked in water for 4 h and then kept in the incubator with moderate humidity at 28 °C for germination. Thereafter, germinated seeds were planted in a plug tray either under artificial light irradiation (LED light model, defined as normal light) or dim light in the greenhouse of Vegetable Research Institute, Guangdong Academy of Agricultural Sciences. Dim light treatment was performed in a black plastic bag covered homemade growth chamber. Light intensity of normal light and of dim light was 8000 LUX and 35 LUX, respectively. Seven-day-old seedlings of wild type and *alc* mutant were used for phenotypic evaluation, fluorescence microscopy, transmission electron microscopy analysis, and high-throughput RNA sequencing.

### Pigment content measurement

To determine the chlorophyll a (Chla), chlorophyll b (Chlb), total chlorophyll (Chl), and carotenoid (Car) contents, wild type and *alc* mutant seedlings grown under normal and dim light conditions were compared. A total of 0.1 g cotyledon from each sample was cut into small pieces and transferred into 10 mL 80% (v/v) acetone and kept in dark until the tissue turned white. Each sample was analyzed in three biological replicates. For each sample, the absorbance was measured at 663, 645, and 470 nm thrice, respectively. Concentrations of Chla, Chlb, Chl, and Car were calculated as described previously (Lichtenthaler, 1987).

### Fluorescence microscopy

For fluorescence microscopy, the abaxial epidermis of cotyledons was used. Chloroplast autofluorescence (red) was captured under Zeiss LSM710 (Germany) confocal microscope with the following settings: excitation at 633 nm, emission at 647–721 nm. Data were analyzed using software ZEN (2010).

### Transmission electron microscopy(TEM)

Cotyledons of wild type and *alc* mutant seedlings grown under dim light conditions and normal light irradiation were analyzed by TEM. All the cotyledon samples were cut into 1–2 mm^2^ sections and fixed in 2.5% glutaraldehyde and 4% paraformaldehyde in phosphate buffer (pH 6.8–7.2) under vacuum for 3 h. After washing with phosphate buffer the samples were fixed in 1% osmium tetroxide (OsO_4_) for 3 h and again washed with phosphate buffer. The samples were dehydrated through a series of ethanol concentration. The samples were infiltrated with an increasing ratio of Spurr’s resin dilutions [25%, 50%, 75%, and 100% (v/v)] to substitute ethanol, and finally embedded in Spurr’s resin. After cutting, the sections were viewed under a HitachiH-7700 (Hitachi) transmission electron microscope.

### RNA extraction

Total RNA was extracted from cotyledons of 7-day-old wild type and *alc* mutant seedlings using Trizol Kit (Promega, USA) according to the manufacturer’s instructions. Extracted RNA was treated with RNase-free DNase I (TaKaRa, Japan) to remove residual DNA. RNA degradation and contamination were monitored on 1% agarose gels. RNA purity was checked using the NanoPhotometer^®^ spectrophotometer (IMPLEN, CA, USA) and RNA integrity was assessed using the RNA Nano 6000 Assay Kit of the Bioanalyzer 2100 system (Agilent Technologies, CA, USA).

### cDNA library construction, high-throughput sequencing and mapping

Cotyledons of wild type and *alc* mutant seedlings grown under normal light conditions were collected for high-throughput sequencing. Each sample was analyzed in three biological replicates. A total of 1 µg RNA per sample was used as input material for the RNA sample preparations. Sequencing libraries were generated using NEBNext^®^ UltraTM RNA Library Prep Kit for Illumina^®^ (NEB, USA) following manufacturer’s recommendations. Library preparations were sequenced on an Illumina Novaseq platform and 150 bp paired-end reads were generated. Thereafter, reads with adaptors, reads with unknown bases, as well as low quality reads were removed to generate clean reads. The remaining high-quality clean reads were mapped to Cucumber (Chinese Long) Reference Genome v2 (http://www.cucurbitgenomics.org/organism/2).

### Quantification of gene expression, GO, and KEGG pathway enrichment analysis

The mapped reads of each sample were assembled using StringTie (v1.3.3b) 17 in a reference-based approach, and featureCounts v1.5.0-p3 18 was used to count the reads numbers mapped to each gene. Fragments Per Kilobase of transcript sequence per Millions base pairs (FPKM) of each gene was calculated based on the gene length and read count mapped to this gene. Differential expression analysis of two groups was performed using the DESeq2 R package (1.16.1) 19. The resulting P-values were adjusted using the Benjamini and Hochberg’s approach for controlling the false discovery rate. Genes with an adjusted P-value (padj) <0.05 and |log2(FoldChange)| > 2 were assigned as differentially expressed genes (DEGs). To functionally annotate the DEGs, Gene Ontology (GO, http://www.geneontology.org/) and Kyoto Encyclopedia of Genes and Genomes (KEGG, http://www.genome.jp/kegg/) annotation of the unigenes were analyzed using clusterProfiler R package.

### Quantitative real-time PCR validation

To confirm RNA-seq results, 12 DEGs were selected for qRT-PCR validation. First strand cDNA synthesis was performed using TransScript All-in-One First-Strand cDNA Synthesis SuperMix for qPCR (Transgen, China) with 1 μg of RNA used for high-throughput sequencing. Quantitative RT-PCR was carried out using 0.2 μg cDNA using PerfectStart Green qPCR Supermix (Transgen, China) according to the manufacturer’s instructions. Reactions were performed and analyzed on CFX96 Real-Time PCR Detection System. Three biological replicates and three technical replicates per sample were performed for each gene. Gene expression was normalized against α‐TUBULIN *(TUA)* gene (Liu et al., 2016). Primers used are listed in **Supplementary Table 1**.

## Results

### Phenotypic characterization of a cucumber *alc* mutant

**Figure 1:**
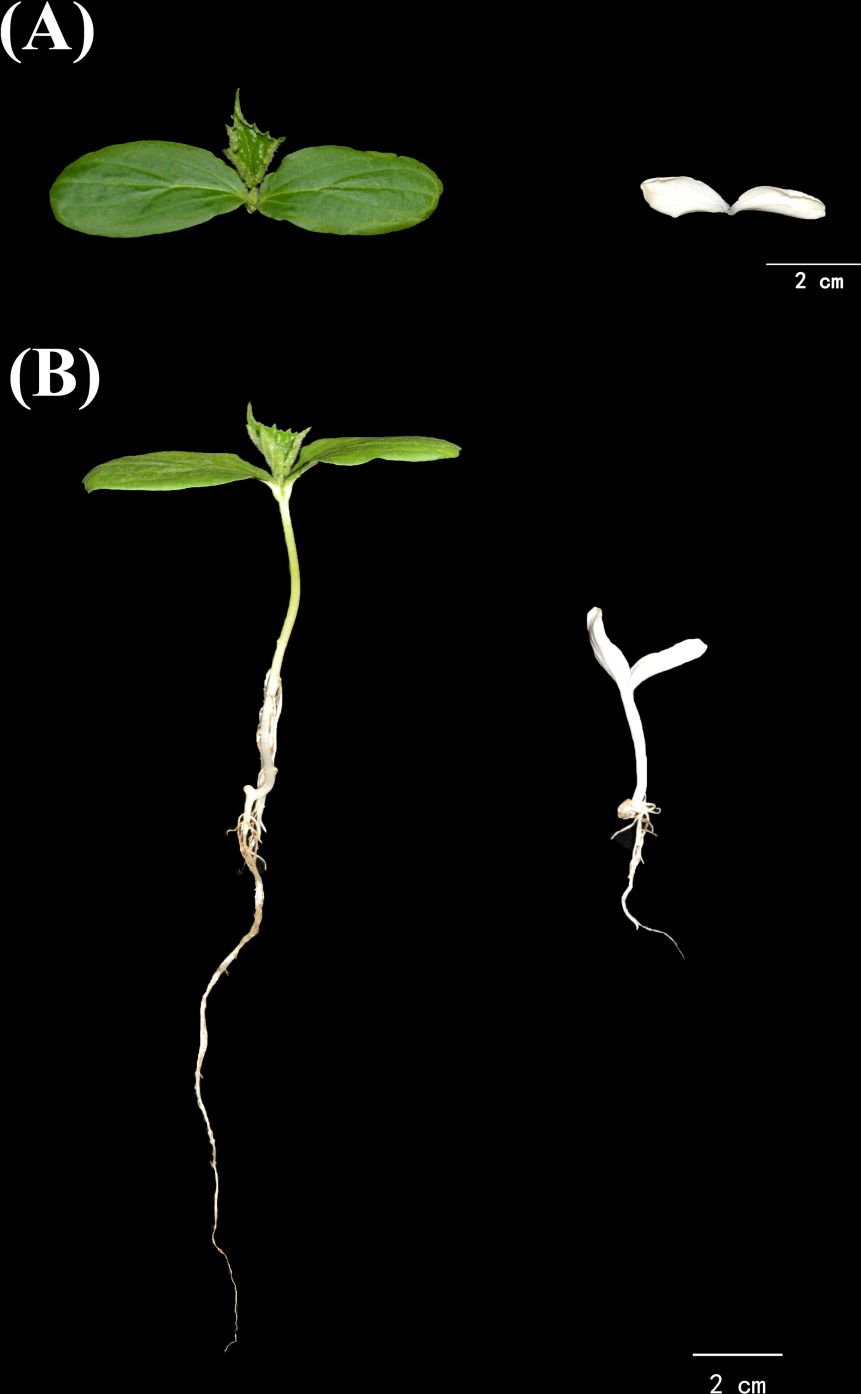
Growth phenotype of *alc* and wild type from the progenies of a cucumber inbred line g32. **(A)** Different cotyledons of *alc* and wild type seedlings. **(B)** Morphological difference between *alc* and wild type seedlings.

We observed a few naturally occurring albino seedlings during the reproduction of cucumber inbred line “g32”, which is a southern China type cucumber. The cotyledons of these seedlings were small and entirely white, with short and white hypocotyl and short primary root (**Figure 1**). The mutant was named as *alc* (albino cotyledon) thereafter. The mutants dried out and died in a few days after emerging from the substrate without growing any true leaves.

**Figure 2:**
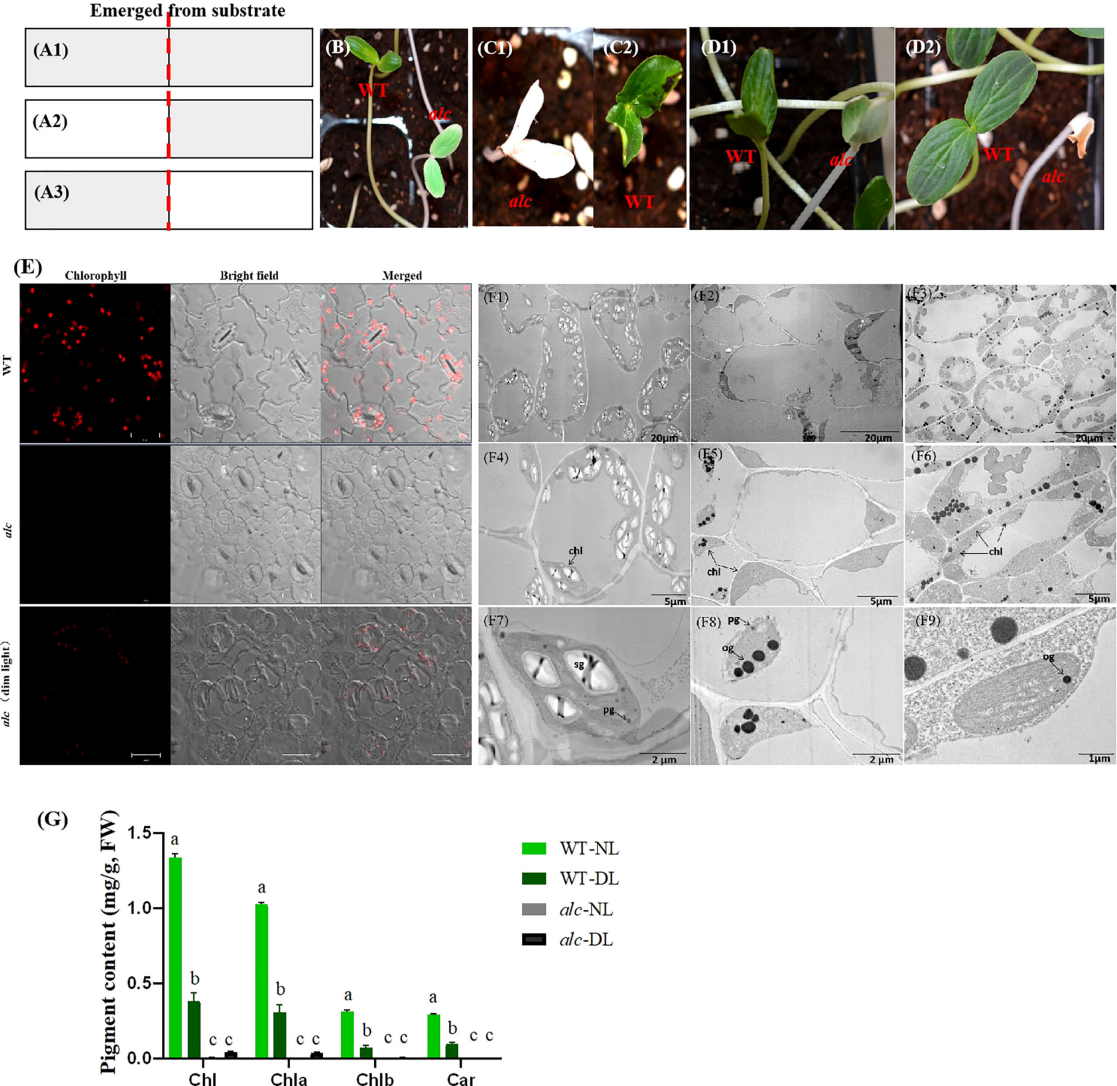
Short-lived chloroplast recovery in *alc* mutant under dim light condition. **(A1–A3)** Schematic illustration of light treatments of WT and *alc* seedlings. A1 Plants were treated with continuous dim light before and after they emerged from substrate. A2 First, plants were treated with normal light irradiation, then after emerging from substrate, they were moved to dim light condition. A3 First, plants were treated under dim light, then after emerging from substrate, they were moved to normal light condition. **(B)** Phenotype of WT and *alc* seedlings under indicated condition that described in a1. **(C)** Phenotype of WT **(C1)** and *alc* **(C2)** seedlings under indicated conditions that described in A2. **(D)** Phenotype of WT and *alc* seedlings under indicated conditions that described in A3. D1 Phenotype of WT and *alc* seedlings exposed to normal light after 5 hours. D2 Phenotype of WT and *alc* seedlings exposed to normal light after 30 hours. **(E)** Fluorescence microscopy images of cotyledon abaxial epidermis of wild type, *alc* mutant cucumber seedlings grown under normal light condition and *alc* mutant seedlings grown under dim light condition. Bar: 20μm. **(F1–F9)** Transmission electron microscopy of cotyledons from *alc* mutant and wild type seedlings. F1, F4 An overview of cotyledon cells of wild type grown under normal light condition. **(F2–F5)** An overview of cotyledon cells of *alc* mutant grown under normal light condition. F3, F6 An overview of cotyledon cells of *alc* mutant grown under dim light condition. **(F7–F9)** Enlarged views of chloroplast ultrastructure of wild type, *alc* mutant grown under normal light condition and *alc* mutant grown under dim light condition, respectively. chl, chloroplast sg, starch granules pg, plastoglobuli og, osmiophilic plastoglobuli. **(G)** Chlorophyll content and carotenoid content of wild type and *alc* mutant grown under normal light and dim light conditions. WT-NL, wild type grown under normal light condition. WT-DL, wild type grown under dim light condition alc-NL, *alc* mutant grown under normal light condition alc-DL, *alc* mutant grown under dim light condition. Chla, chlorophyll a; Chlb, chlorophyll b; Chl, total chlorophyll.

It was noteworthy that occasionally cotyledons of a few *alc* mutants that were still inside the shell showed subtle greenish; this led us to propose that the occurrence of greenish cotyledons in *alc* mutants may be affected by light. Therefore, we performed three sets of experiments to validate this hypothesis (**Figure 2A**). In the first experiment, all the seeds were grown under dim light conditions at all times. After emerging from the substrate, *alc* mutants presented creamy green cotyledons with complete white hypocotyl, while wild type seedlings showed yellowish-green cotyledons and hypocotyl (**Figure 2B**). In the continually dim light environment, both *alc* mutant and wild-type plants spindled and died without growing first true leaf. In the second experiment, after the seeds emerged from the substrate, they were first allowed to grow under normal light conditions until we were able to distinguish between *alc* mutant and wild-type plants. We then transferred them to dim light conditions and observed that the cotyledons of *alc* mutants remained whitish, without turning to cream green (**Figures 2C1, C2**). In the last experiment, the seeds were grown under dim light conditions until seedlings emerged from substrate, and then they were exposed to normal light condition (**Figure 2A3**). After 5 h of light exposure, the green color in the cotyledon of *alc* mutant started to degrade (**Figure 2D1**). The cotyledon shrunk and dried out after exposure to light for 30 h (**Figure 2D2**). Therefore, we conclude that *alc* is a light sensitive albino mutant. Normal light irradiation is a lethal factor for *alc* mutant and, the damage caused by light is irreversible.

Most of the albino phenotypes in other plants lack chlorophyll and carotenoids. Thus, we examined the chlorophyll and carotenoid content of cotyledons from both wild type and *alc* mutant seedlings that grew under normal light and dim light conditions. Fluorescence microscopy revealed that chlorophyll fluorescence in wildtype was more intense than that observed in *alc* mutants grown under dim light conditions (**Figure 2E**; presented in red). As expected, no chlorophyll fluorescence was detected in *alc* mutants that grew under normal light conditions. Chlorophyll content was measured based on the fluorescence intensity (**Figure 2G1**). No carotenoid was detected in *alc* mutants grown under normal light conditions; however, a small amount, 0.03 mg/g (fresh weight), was detected in *alc* mutants grown under dim light conditions (**Figure 2G2**).

Since most of the chlorophyll content in plants are in chloroplast, we further investigated the chloroplast ultrastructure in the cotyledons of wild type and *alc* mutant seedlings that grew under both light and dim light conditions using TEM. In the cotyledons of wild type seedlings, we observed numerous well-developed, crescent-shaped chloroplasts with stroma thylakoids, grana thylakoids, starch granules, and plastoglobuli within the membranes **Figures 2F1, F4, F7**). In contrast, the chloroplasts in the *alc* mutant decreased dramatically in number and showed abnormal shapes that lacked stroma and grana thylakoids, but contained osmiophilic plastoglobulis in the inner membrane system (**Figures 2F2, F5, F8**). The *alc* mutants that grew under dim light conditions comprised stroma thylakoid as well as osmiophilic plastoglobulis (**Figures 2F3, F6, F9**).

To summarize, the above results indicated that the chloroplast development was impaired in *alc* mutants grown under normal light conditions during seedling development. Moreover, normal light may be lethal to the *alc* mutant because it interrupts thylakoid biogenesis, as observed by the presence of thylakoid in the *alc* mutant grown in dim light conditions but not in those grown under normal light conditions.

### Inheritance model of the *alc* mutant

As the *alc* mutant died in the seedling stage, we could not obtain homozygous seeds. Therefore, we considered the mutant parental line “g32” as F_1_ and used its self-pollinated seeds to investigate the inheritance pattern of the albino phenotype. We planted a total of 123 seeds from the self-pollinated “g32” cucumber and put them under normal light conditions, of which 116 seeds germinated successfully (germination rate 94.3%). Thirty-two and eighty-four germinated seedlings showed albino and wild type phenotype, respectively. Chi-square analysis (χ^2^ = 0.414, *p* = 0.520) indicated that the segregation ratio between albino and wild type was 1:3. Therefore, the albino trait is likely controlled by a single recessive nuclear gene.

### Transcriptome profiling and identifying differentially expressed genes (DEGs) between the *alc* mutant and wild type seedlings

**Figure 3:**
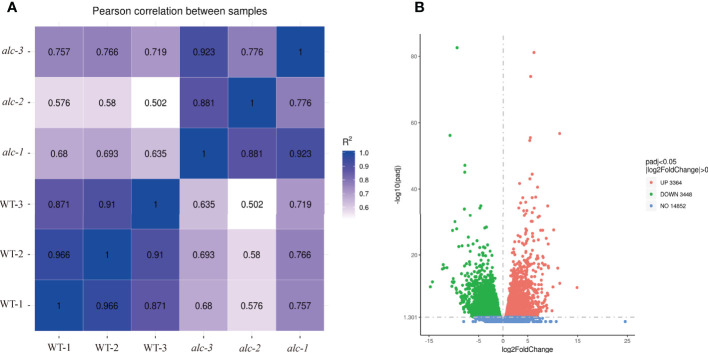
Diagrams illustrating correlations/distances among transcriptomes and the number of differentially expressed genes in *alc* mutant and wild type seedlings. **(A)** Correlation matrix and cluster dendrogram of the whole dataset of the mapped reads. The analysis was performed by comparing the values of the entire transcriptome of all two samples with three biological replicates. Correlation analysis (coefficients R^2^) and hierarchical cluster analysis were performed using R software. Dark blue color indicated a stronger correlation and light blue weaker (R^2^). **(B)** Volcano plot showing DEGs between *alc* mutant and wild type. X-axis represented log2(Fold Change) and y-axis represents -log10 (padj). Red, green and blue dots represented up-regulated, down-regulated and not DEGs, respectively.

**Figure 4:**
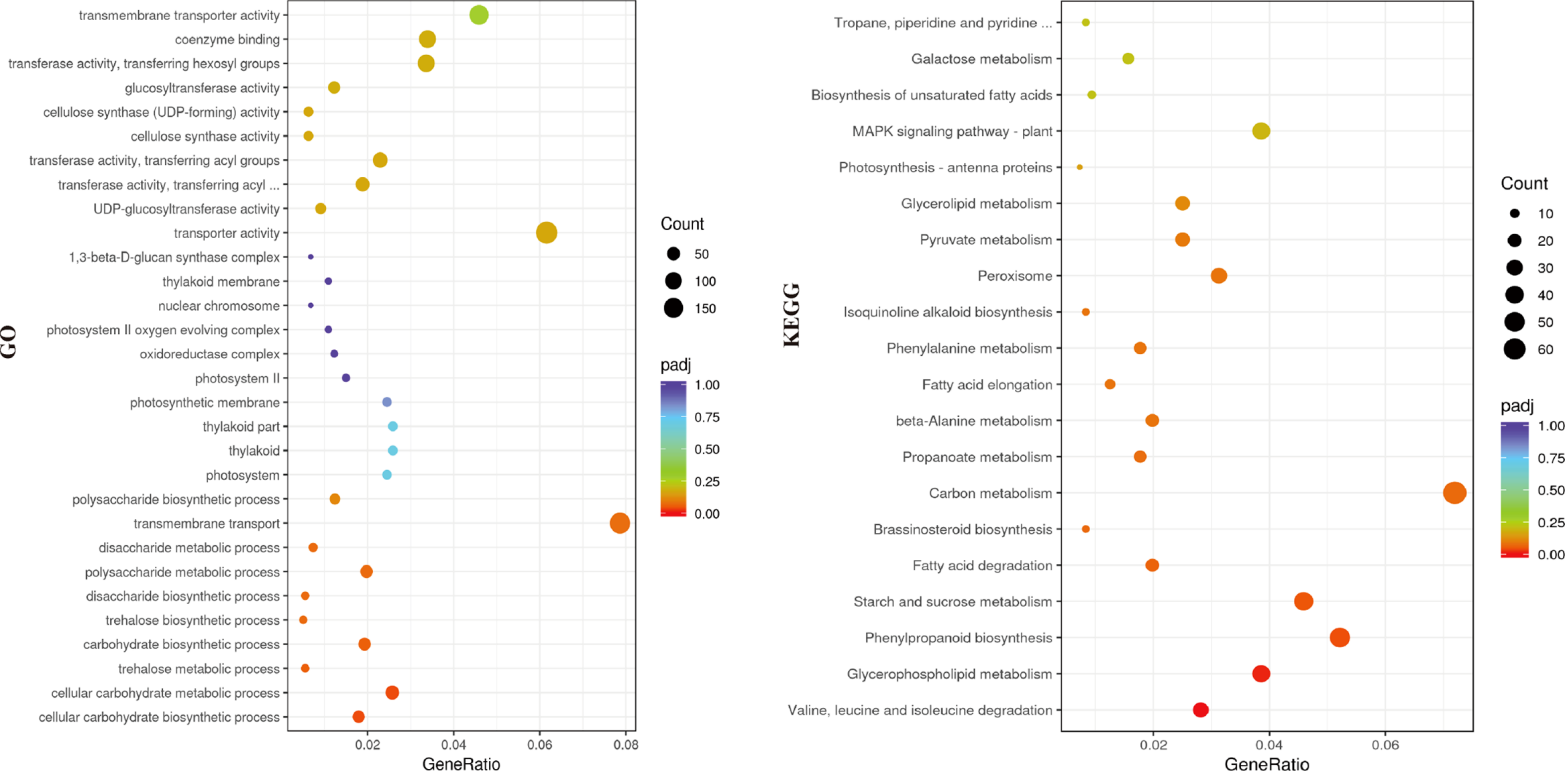
Genes enriched in different categories in the GO (left) and KEGG (right) analysis. X-axis represented the gene ratio of enriched genes among the background genes in different categories and y-axis represented the GO or KEGG terms. A high padj-value was represented by red, and a low value represented by purple. The size of the bubble represented number of genes annotated to each term.

The transcriptomes of cotyledons from the *alc* mutant and wild type seedlings were examined by RNA-seq, each with three biological replicates. Overall, 97,869,948 to 123,516,412 clean reads were obtained after filtering low quality reads. After mapping to the cucumber reference genome 9930 v2 (Huang et al., 2009; Li et al., 2011), 21,664 transcripts were identified. High correlation coefficients among the replicates demonstrated the consistency of the transcriptional changes within each sample (**Figure 3A**). In total, 1,256 genes were upregulated and 1,584 were downregulated in the *alc* mutant compared to the wild type cotyledons (|log2FC| ≥ 2) (**Figure 3B**; **Supplementary Table 2**). Based on the annotation, DEGs were annotated using GO and KEGG pathway to identify the significantly enriched biological processes and pathways between the *alc* mutant and wild type. In total, 2,175 DEGs were classified into 814 GO terms belonging to three categories: biological process, cellular component, and molecular function (**Supplementary Table 3**). Cellular carbohydrate biosynthetic process (GO:0034637, p=0.00010) and cellular carbohydrate metabolic process (GO:0044262, p=0.00017) were the most significantly enriched biological processes (**Supplementary Figure 1**). As shown in **Figure 4**, 171 and 196 DEGs were identified as transmembrane transport (GO:0055085) and transporter activity (GO:0005215), respectively, which were among the most enriched GO terms. (**Supplementary Table 3**). A total of, 957 DEGs were assigned to 110 KEGG pathways (**Supplementary Table 4**), among which, top 20 enriched KEGG pathways are illustrated in **Figure 4**. Carbon metabolism (KEGG: csv01200), phenylpropanoid biosynthesis (KEGG: csv00940), and starch and sucrose metabolism (KEGG: csv00500) pathways contained 69, 50, and 44 DEGs, respectively (**Supplementary Table 4**).

### Differentially expressed genes involved in chlorophyll metabolism

**Figure 5:**
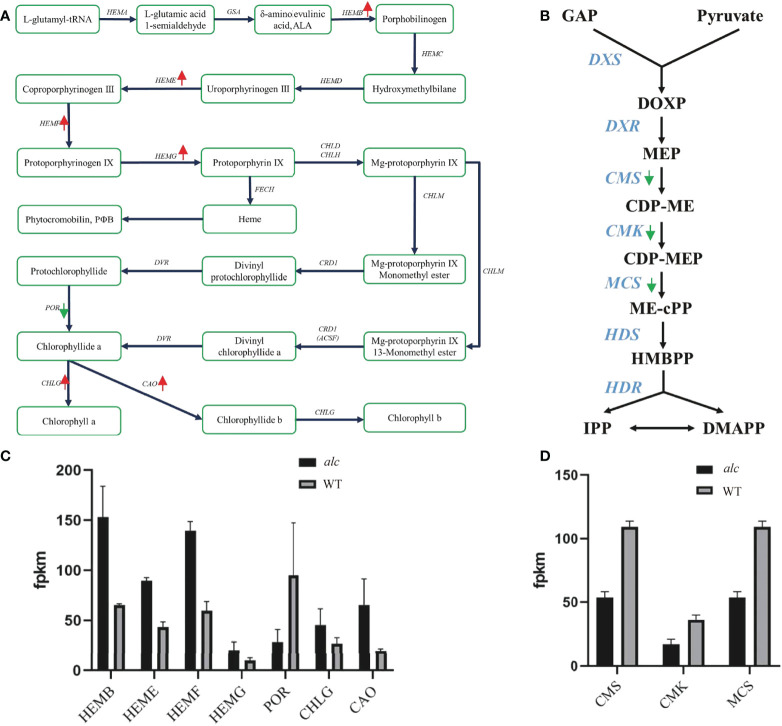
Differential expression of genes involved in chlorophyll metabolism and Methylerythritol 4-phosphate (MEP) pathway. **(A, B)** Diagram showing major genes in chlorophyll metabolism and MEP pathway, respectively. Red arrows indicated up-regulated genes and green arrows indicated down-regulated genes in *alc* mutant. **(C, D)** Expression profile of DEGs in chlorophyll metabolism and MEP pathway achieved by RNA-seq. The mean FPKM (fragments per kilo base of transcript per million mapped reads) values for the DEGs were calculated from three biological replicates for each genotype. Error bars indicated standard deviations. GAP, glyceraldehyde-3-phosphate; DOXP, 1-deoxy-D-xylulose-5-phosphate; MEP, 2-C-methyl-D-erythritol-4-phosphate; CDP-ME, 4-diphosphocytidyl-2-C-methyl-D-erythritol; CDP-MEP, 4-diphosphocytidyl-2-C-methyl-D-erythritol-2-phosphate; ME-cPP, 2-C-methyl-D-erythritol-2,4-C-cyclodiphosphate; HMBPP, 1-hydroxy-2-methyl-2-(E)-butenyl-4-diphosphate; IPP, isopentenyl diphosphate DMAPP, dimethylallyl diphosphate.

Porphyrin and chlorophyll metabolism (KEGG: csv00860) was a significantly enriched pathway in KEGG analysis. Many key enzymes involved in this pathway showed distinct expression profile between the *alc* mutant and the wild type. The expression of most genes, including *HEMB* (Csa2G401270), *HEME* (Csa4G082410, Csa5G218840), *HEMF* (Csa4G056670), *HEMG* (Csa6G007980), CHLG (Csa4G311220), and CAO (Csa6G385090), was slightly higher in the *alc* mutant than in the wild type (**Figure 5A**; **Supplementary Table 5**). However, POR (Csa4G638340) was downregulated in the *alc* mutant, presenting an opposite expression pattern to that of other genes (**Figure 5A**; **Supplementary Table 5**).

### Differentially expressed genes involved in methylerythritol 4-phosphate (MEP) pathway

The methylerythritol 4-phosphate (MEP) pathway is mainly involved in the production of isoprenoid precursors; isopentenyl diphosphate (IPP) and dimethylallyl diphosphate in photosynthetic eukaryotes (Cordoba et al., 2009). As shown in **Figure 5B**, there were at least seven key enzymes involved in the MEP pathway. Four candidate genes encoding CMS (Csa3G113320 and Csa4G049620), CMK (Csa1G600780), and MCS (Csa4G049620) were downregulated in the *alc* mutant (**Supplementary Table 5**).

### Chloroplast development-related genes

Chloroplast-localized FtsH proteins are crucial for the biogenesis of thylakoid membranes (Wagner et al., 2012). However, we observed negligible expression of the two *FtsH genes* (Csa6G504470 and Csa6G504480) in the *alc* mutant (**Supplementary Table 2**). Some pentatricopeptide repeat (PPR) proteins are involved in plastid gene expression and can also affect chloroplast development (Myouga et al., 2010). The expression of *PPR* gene (Csa5G189930) in the *alc* mutant was more than 3.5-fold higher than that in the wildtype (**Supplementary Table 2**).

### Thylakoid related genes were affected in *alc* mutant

**Figure 6:**
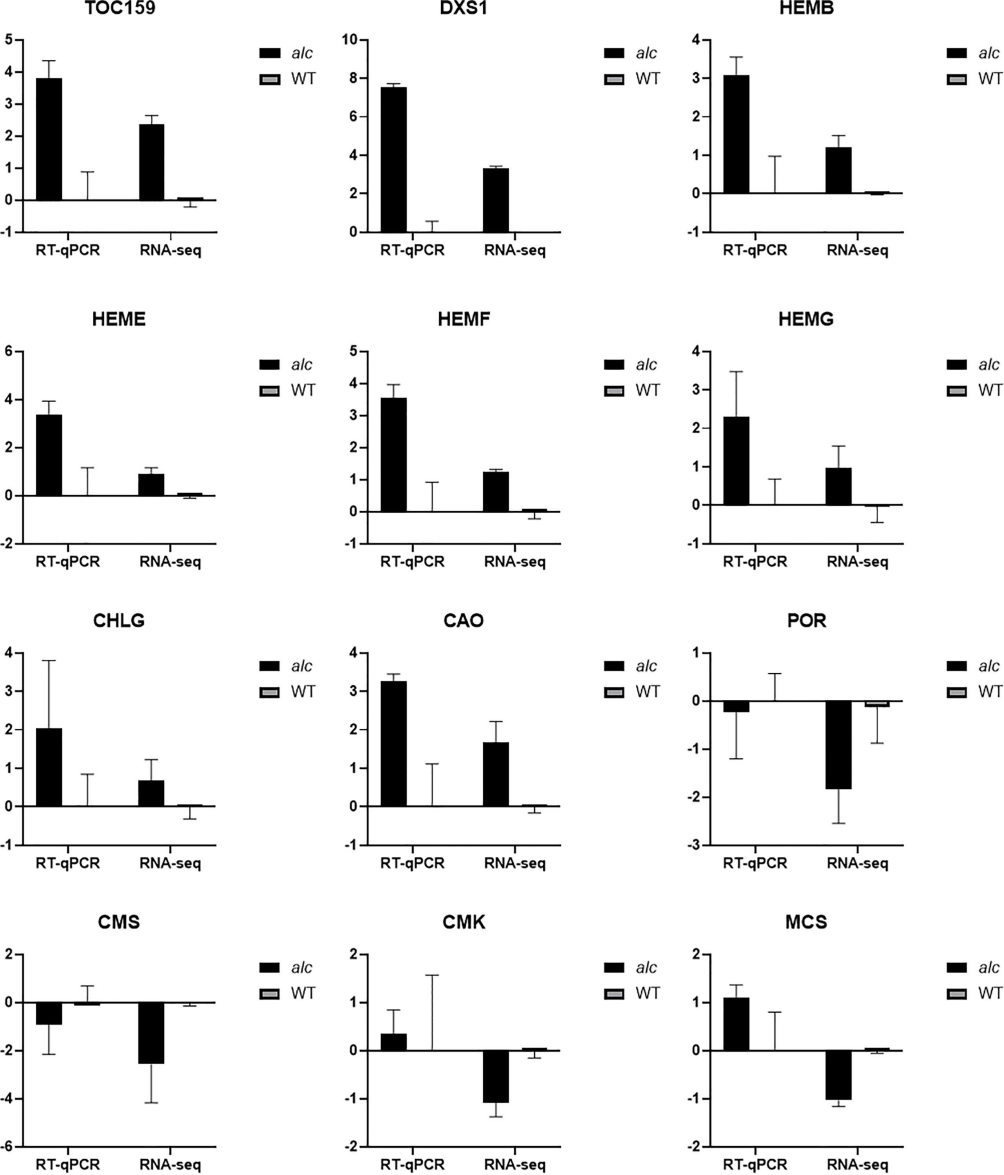
Expression profile of selected differentially expressed genes between *alc* mutant and wild type from RNA-seq result achieved by qRT-PCR. X-axis represented gene name and y-axis represented relative expression (–ΔΔCt) value of each gene. Data are shown as means (n = 3). Error bars indicated standard deviations.

Nineteen DEGs involved in thylakoid related functional activities, including thylakoid (GO:0009579), thylakoid part (GO:0044436), and thylakoid membrane (GO:0042651), were highlighted (**Figure 6**; **Supplementary Table 3**). Three *PsbPs* genes (Csa2G030040, Csa1G088470, and Csa1G181310) were upregulated in the *alc* mutant, while 16 other genes, namely, *PsbR* (Csa4G064020), *PsaG/PsaK* (Csa3G060980, Csa6G525340), *PsbO* (Csa6G488340), *PsaD* (Csa3G147780), *PsaH* (Csa3G483830), *PsbP* (Csa4G063440), *PsaE* (Csa2G079660), *PsbY* (Csa5G592810), *PsbW* (Csa7G378440), *PsaN* (Csa6G483300), *PsbQ* (Csa1G066480, Csa3G414060), *PsbX* (Csa1G595840), *PsaF* (Csa1G714680), and *PetM* (Csa7G075020) were downregulated in the *alc* mutant (**Supplementary Table 2**).

### Homologous genes from albino mutants in other plant species

Previous studies have reported that mutations of FRUCTOKINASE-LIKE PROTEIN in barley and rice (Qin et al., 2015; Lv et al., 2017; He et al., 2018), *RPL21c* (chloroplast 50S ribosomal protein L21) in Arabidopsis and rice (Yin et al., 2012; Lin et al., 2015), *EMB* (embryo-defective) in Arabidopsis (Huang et al., 2009; Liang et al., 2010; Ye et al., 2017; Chen et al., 2018), *PDS3* (phytoene desaturase) in Arabidopsis (Qin et al., 2007), *TOC159* (Translocase of chloroplast 159) in Arabidopsis (Kakizaki et al., 2009; Shanmugabalaji et al., 2018), *DXS1* (1-deoxy-D-xylulose-5-phosphate synthase 1) in tomato (Garcia-Alcazar et al., 2017), and *PurD* (phosphoribosylamine–glycine ligase) in rice (Zhang et al., 2018) can cause albinism. The coding sequences (CDSs) of the above mentioned homologous genes of *alc* mutant and wild type were compared using our transcriptome data. However, no variant was found among these genes in the*alc* mutant and wildtype. Among these genes, only *TOC159* (Csa4G001670) and *DXS1* (Csa3G114510) showed more abundant expression in *alc* mutant than in the wild-type (**Supplementary Table 6**).

### Validation of DEG expression by RT-qPCR

Twelve genes including seven genes involved in chlorophyll metabolism, three genes from the MEP pathway, *TOC159*, and *DXS1* were selected for RT-qPCR verification (the information of the genes is listed in **Supplementary Table 1**). The relative expression (–ΔΔCt) of each gene was calculated using corresponding wild type gene expressions as control. The correlation between the relative expression value and RNA-seq result (log2FoldChange) of the *alc* mutant was determined. The correlation between RT-qPCR and RNA-seq data was 0.9780 (P < 0.0001, ^****^), indicating the reliability of our RNA-seq results.

## Discussion

Albinism occurs among different living organisms ranging from human beings to animals as well as higher plants (Kamaraj and Purohit, 2014; Galante Rocha de Vasconcelos et al., 2017; Le Ret et al., 2018). In the present study, some albino seedlings were observed in the progenies of a self-pollinated cucumber fruit during seed increase period of a cucumber inbred line named “g32”. The mutant was named *alc*, which presented white cotyledons and hypocotyl, and died before developing first true leaf under normal light conditions. In most studies, albino plants were caused by lack of chlorophyll and impaired chloroplast development (Liang et al., 2010; Zhu et al., 2015; Le Ret et al., 2018). Consistent with most albino mutants, lack of chlorophyll and defective chloroplast development was also observed in the *alc* mutant (**Figure 2**). In wild-type seedlings, ultrastructure of chloroplast was well presented with compactly arranged chloroplasts, while few or even no chloroplasts were observed in albino seedlings. Furthermore, an abnormal chloroplast ultrastructure lacking starch granules and thylakoids, but with osmiophilic plastoglobuli was observed in the *alc* mutant (**Figure 4**). Osmiophilic plastoglobuli generally appear as a result of the degradation of thylakoid membranes under stress (Wang et al., 2016) Therefore, we propose that irradiation with normal light might act as an abiotic stress cue for *alc* mutant, leading to the degradation of thylakoid membranes.

Interestingly, we found that the cucumber *alc* mutant differed from the reported *fln1, rpl21c, emb, pds3, toc159, dxs1, and purd* albino mutants as this mutant presented green cotyledons under dim light conditions. Additionally, we checked the homologous gene sequence of these albino related genes in cucumber, but found no variation within the coding sequences of the *alc* mutant and wild type, suggesting that *alc* might be a novel light sensitive albino mutant. Similar phenotype was reported in *pap7-1* albino mutant of Arabidopsis (Grübler et al., 2017). *Pap7-1* mutant had an albino cotyledon and died grown under light conditions; however, under dark conditions, *pap7-1* could grow when supplied with sucrose supplemented medium (Grübler et al., 2017). However, the molecular mechanism describing the exact nature of *pap7-1* is still unknown.

Since all the seeds were planted under the same culture condition, we could exclude the possible of environmental influences on the albino phenotype of the *alc* mutant. Genetic analysis revealed that this albino phenotype was controlled by a recessive locus. Phenotypic evaluation of the previous generation of “g32” was also performed but albino seedlings were not observed (data not shown), confirming the recessive inheritance of this albino allele. Albinism is not a desired phenomenon in plant breeding since it could affect plant growth as well as production. However, this *alc* mutant is of great importance to us for detection of new genes controlling plastid development.

Transcriptome analysis has been extensively applied to identify major genes and dissect regulatory networks involved in albinism by using different albino mutants (Shi et al., 2017; Li et al., 2017; Ma et al., 2018). In our study, transcriptome analysis revealed that the expression of genes involved in chloroplast development, chlorophyll metabolism, MEP pathway, and other genes, such as glutathione S-transferase, was altered in the *alc* mutant. This transcriptional modification suggests that these genes play important roles for the albino phenotype of the *alc* mutant.

Light plays a crucial role in plant development. In light-sensitive mutants, a sudden increase in ROS production would occur under excessive light and induce oxidative damage leading to leaf bleaching. Arabidopsis *ch1* is a Chlb deficient mutant and is devoid of photosystem II (PSII) Chl-protein antenna complexes. Thus, oxidizing side of PSII is impaired and *ch1* is sensitive to photooxidative stress (Havaux et al., 2007). Nearly all DEGs in the Photosynthesis-antenna proteins pathway (**Supplementary Table 4**, KEGG: csv00196) were downregulated in the *alc* mutant, indicating a weak functionality of core reaction center. Peroxisomes are organelles that contribute to the reduction of oxidative stress (del Río et al., 2006). Most of genes in Peroxisome pathway (24 out of 30) (**Supplementary Table 4**, KEGG: csv04146) were upregulated in the *alc* mutant. Under normal light irradiation for wild-type but excessive for *alc*, the *alc* might produce more ROS and lead to bleaching and finally died as *ch1* (Ramel et al., 2013). Since *pap7-1* mutant could be arrested under very dim light (Grübler et al., 2017), we tried to culture the mutant under dim light condition but failed. Further studies are needed to confirm whether the morphology and flowering of the mutant are similar to that of *pap7-1* grown under sucrose supplemented medium and dim light conditions.

Three genes involved in chloroplast development were found to show different transcript levels between the *alc* mutant and wild type. Two of these genes belong to FtsH family, which is essential for chloroplast development. For example, both *ftsh1 ftsf5* and *ftsh2 ftsh8* double mutants developed white seedlings with disrupted chloroplast development (Zaltsman et al., 2005). Significantly fewer *FstH* transcripts were detected in the *alc* mutant as compared to those in wild-type cucumber. E*ntratricopeptide Repeat Protein Pigment-Defective Mutant2 (PDM2)*, that encodes a PPR protein, is required for chloroplast development by regulation of plastid gene expression (Du et al., 2017). We detected very low expression of a *PPR* gene in the *alc* mutant. The extremely low expression of chloroplast related genes might impact chloroplast development and result in an albino phenotype. Key genes involved in chlorophyll metabolism, the MEP pathway, and thylakoid function were also affected in the *alc* mutant. Many genes in the chlorophyll metabolism pathway, including *HEMB*, *HEME*, *HEMF*, *HEMG*, and *CHLG* were slightly up regulated in the *alc* mutant. The increased expression of these genes was also observed in other albino mutants. Most of the chlorophyll-biosynthesis related genes, such as *HEMC*, *HEME* and *CHLG* were upregulated in white leaves compared with those in green leaves of *Ananas comosus* var. *Bracteatus* (Li et al., 2017). Similar results were observed in the wheat *mta* albino mutant where the expression of *HEME* was upregulated (Shi et al., 2017). The decreased expression of most genes might be regulated by a feedback mechanism. *POR*, a key light-dependent enzyme, is essential to chlorophyll biosynthesis where it catalyzes protochlorophyllide to chlorophyllide (Yang and Cheng, 2004; Zhang et al., 2019). POR is crucial for plant growth and development because *por* mutant and POR RNAi line displayed reduced chlorophyll content and severe photoautotrophic growth defects (Kim and Apel, 2012; Paddock et al., 2012). The expression level of POR was low in the *alc* mutant, the *mta* albino wheat mutant (Shi et al., 2017), and the complete white leaves of *Ananas comosus* var. *Bracteatus* (Li et al., 2017), indicating that the downregulation of POR might have an impact on chlorophyll synthesis in the *alc* mutant. The MEP pathway is essential for the biosynthesis of photosynthesis-related compounds, such as carotenoids, chlorophylls, gibberellins, and abscisic acid, which are of vital importance for plant development and metabolism (Flores-Perez et al., 2008). The mutation of genes in the pathway impaired the biosynthesis of these compounds, disrupted chloroplast development, and resulted in abnormal plant morphology, especially in leaf color (Xing et al., 2010; Chandran et al., 2016). IspD (CMS), IspE (CMK), and IspF (MCS) are the third, fourth and fifth enzymes in the MEP pathway, respectively (**Figure 5B**). Related mutants possessed yellow or albino leaves with arrested development of chloroplasts (Hsieh et al., 2008; Chen et al., 2018; Huang et al., 2018). In this study, the expression of CMS, CMK and MCS in the *alc* mutant was lower than that in the wildtype. The low-level expression may contribute to the disruption of chloroplast development. Thylakoid related genes corresponded to reaction centers of PSI and PSII where photochemical reactions occur and convert light energy into chemical energy. Almost all genes related to thylakoid were downregulated in the *alc* mutant, demonstrating the decrease of photosynthesis viability.

Isocitrate lyase and malate synthase are two enzymes unique to the glyoxylate cycle, which is considered essential for postgerminative growth and seedling establishment (Eastmond et al., 2000). Under normal light conditions, the enzyme activities in the glyoxylate cycle decreased rapidly as seedlings become photosynthetic, whereas those under dark conditions maintained a continuously high level of enzyme activity (Trelease et al., 1971; Becker et al., 1978; He et al., 2016). In this study, the abundance of isocitrate lyase (Csa2G420990) and malate synthase (Csa1G050360) was higher in the mutant than in the wildtype (**Figure 6**). The wild-type exhibited autotrophic growth with a high activity of peroxidase (Csa4G285740) and chlorophyll A-B binding protein (Csa3G664560), while the *alc* mutant was unable to perform photosynthesis due to the lack of functional chloroplasts. Glutathione S-transferase have been reported to mainly function in response to biotic and abiotic stresses, such as oxidative stress (Horváth et al., 2015), temperature stress (Roxas et al., 1997; Liang et al., 2018) and different pathogen invasion (Sytykiewicz et al., 2014; Gong et al., 2018; Ducker et al., 2019). The high concentration of glutathione S-transferase can result in decreased chlorophyll content (Jiang et al., 2010). When the *alc* mutant is exposed to light, which is lethal rather than beneficial, it expressed (Csa4G304240, Csa4G303170) (**Figure 6**) more glutathione S-transferase than the wild type; this might also promote chlorophyll degradation.

In conclusion, a cucumber albino mutant *alc* was cytologically, genetically and transcriptomically characterized in this study. Our results demonstrated that the albino phenotype of the mutant was mainly due to the disability in chlorophyll synthesis and chloroplast development, which resulted in no chlorophyll content in the cotyledons and finally seedlings died as not being able to photosynthesize. In this study, we could not determine the causal mutation of *alc*. However, Bulk Segregant Analysis (BSA), using albino and green seedling pools along with map-based cloning would be useful to finely mapping the mutated gene in future studies.

## Data availability statement

The RNA-seq datasets presented in this study can be accessed through National Center for Biotechnology Information (NCBI) BioProject database under accession number PRJNA685868.

## Author contributions

JY, BL, YL, and BJ: conceptualization and writing—review and editing; JY, ZC, LC, ZL, MW, and WL: formal analysis and investigation; JY: writing—original draft preparation. All authors have read and approved the final manuscript.

## Funding

This study was supported by Key-Area Research and Development Program of Guangdong Province (2020B020220001), The Discipline Team Construction Project of GDAAS (202103TD), the Training Plan for Young and Middle-aged Discipline Leaders of GDAAS (R2020PY-JG003) and Talent Introduction Plan of GDAAS (R2021YJ-YB2004). The funding bodies have no role in the study design, data analysis and interpretation, and manuscript writing, but just provide the financial supports.

## Acknowledgments

The authors thank associate professor Yuhui Wang from College of Horticulture at Nanjing Agricultural University for her assistance in language editing.

## Conflict of interest

The authors declare that the research was conducted in the absence of any commercial or financial relationships that could be construed as a potential conflict of interest.

## Publisher’s note

All claims expressed in this article are solely those of the authors and do not necessarily represent those of their affiliated organizations, or those of the publisher, the editors and the reviewers. Any product that may be evaluated in this article, or claim that may be made by its manufacturer, is not guaranteed or endorsed by the publisher.

## Supplementary material

Supplementary Table 1 Primers used for qRT-PCR validation.

Supplementary Table 2 Up-regulated and down-regualted genes in albino mutant.

Supplementary Table 3 GO enrichment analysis of differentially expressed genes between albino mutant and wild type.

Supplementary Table 4 KEGG analysis of differentially expressed genes between albino mutant and wild type.

Supplementary Table 5 Differentially expressed genes in chlorophyll metabolism and MEP pathway between albino mutant and wild type.

Supplementary Table 6 Differentially expressed genes of reported albino genes between albino mutant and wild type.

Supplementary Figure 1 Histogram of GO enrichment analysis.

Supplementary Figure 2 A heat map showing expression patterns of genes related to chlorophyll metabolism, chloroplast formation and Methylerythritol 4-phosphate (MEP) pathway.
