# Supplementary material for: Cytological, genetic and transcriptomic characterization of a cucumber albino mutant
Source: Front Plant Sci. 2022 Oct 20;13:1047090. doi: 10.3389/fpls.2022.1047090 (PMC9630852; doi:10.3389/fpls.2022.1047090)
Supplement: Supplementary Table 1 — Primers used for qRT-PCR validation. [file Table_2.doc]

| Gene Name | Gene ID | Forward primer (5′ → 3′) | Reverse primer (5′ → 3′) |
| --- | --- | --- | --- |
| TOC159 | Csa4G001670 | GGAAGATGAGGAGGAGGACG | ACATCACCCGAATTCCCACT |
| DXS1 | Csa3G114510 | GATCCCAAAACAGGCAAGCA | GCAACGTTGTGGGAACTTCT |
| HEMB | Csa2G401270 | AGCTCGAGATGTTGGTGTCA | GGCCATCAGAAGAGTACGGA |
| HEME | Csa4G082410 | TGGGATGGAGATCTTCGAGC | TGGGCGACTAACAGGATCTC |
| HEMF | Csa4G056670 | TTTGCGACGCCATTGAGAAA | CATTAACGCCAGCCTTCTCC |
| HEMG | Csa6G007980 | ACCAGAGGTCCAGTGCTTAC | TGGGATTTGTAGGCACCAGT |
| CHLG | Csa4G311220 | TGGGTGTTGCTTCTAGGAGG | GCTCCAAGCGCAAAATTTCC |
| CAO | Csa6G385090 | TCATCCCCGCTTGAAGAACT | CGTATGCATCCAGGATTGCC |
| POR | Csa4G638340 | TGGTGCAATAAGGGCTCAGA | AGGCTCCAGTGATCACAACA |
| CMS | Csa3G113320 | GTCATTCTGTTGGCTGGTGG | GGGATCGCAAACCACAATGA |
| CMK | Csa1G600780 | TTCACATGGCTCTCAAGGGT | ATCCCGGTCCACTTCATCAG |
| MCS | Csa4G049620 | GAAGGGTGCTGCATCATCTG | CAGATTCACGACAGAGGGGT |

Supplementary Table 1. Primers used for qRT-PCR validation
