# Supplementary material for: Cytological, genetic and transcriptomic characterization of a cucumber albino mutant
Source: Front Plant Sci. 2022 Oct 20;13:1047090. doi: 10.3389/fpls.2022.1047090 (PMC9630852; doi:10.3389/fpls.2022.1047090)
Supplement: Supplementary Figure 1 — Histogram of GO enrichment analysis. [file Table_1.doc]

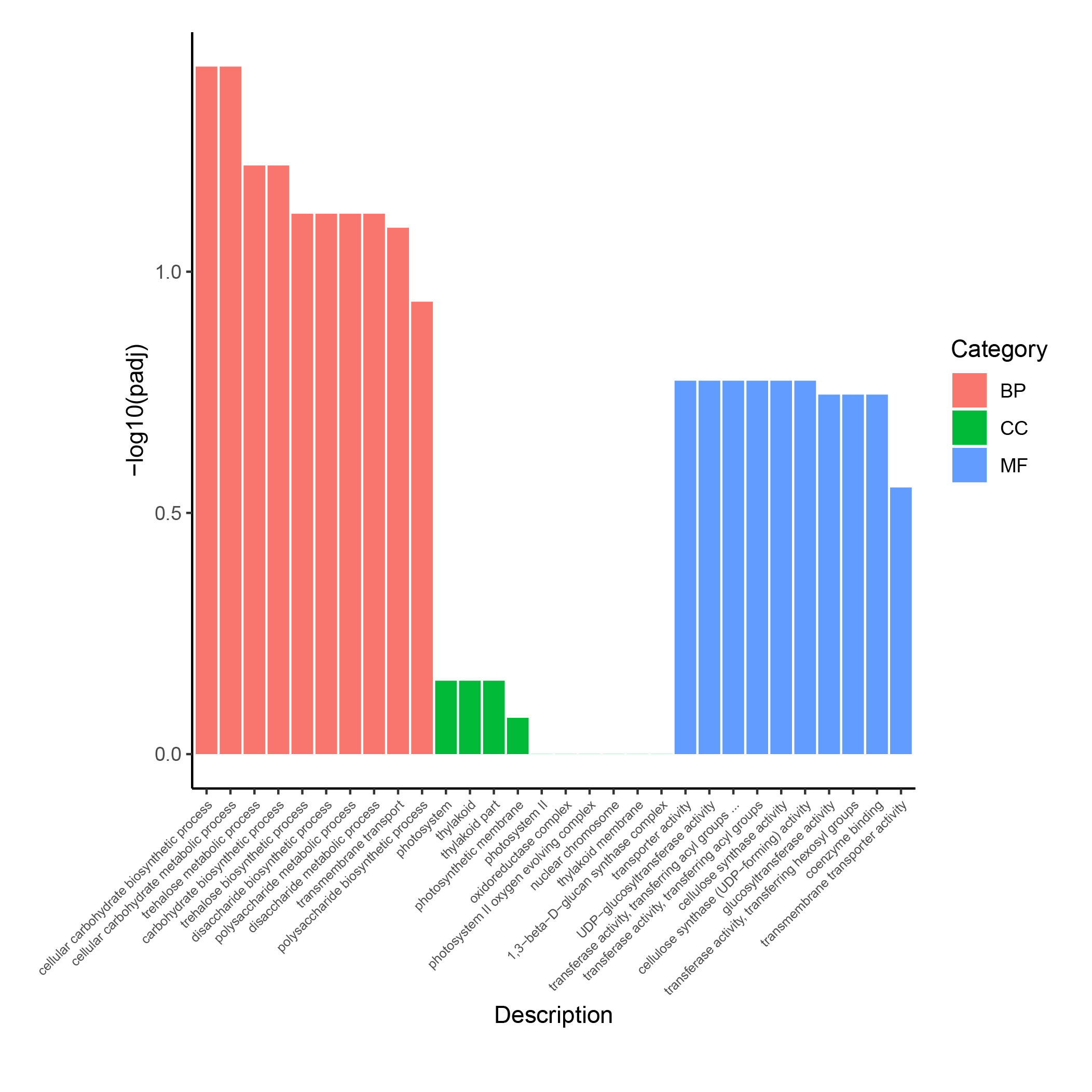


**Supplementary Figure 1. Histogram of GO enrichment analysis.** X-axis indicates GO terms and Y-axis indicates significance of GO enrichment, presented as -log10(padj). BP, biological process; CC, cellular component; MF, molecular function.
