# Supplementary figures and images for: Cytological, genetic and transcriptomic characterization of a cucumber albino mutant
Source: Front Plant Sci. 2022 Oct 20;13:1047090. doi: 10.3389/fpls.2022.1047090 (PMC9630852; doi:10.3389/fpls.2022.1047090)

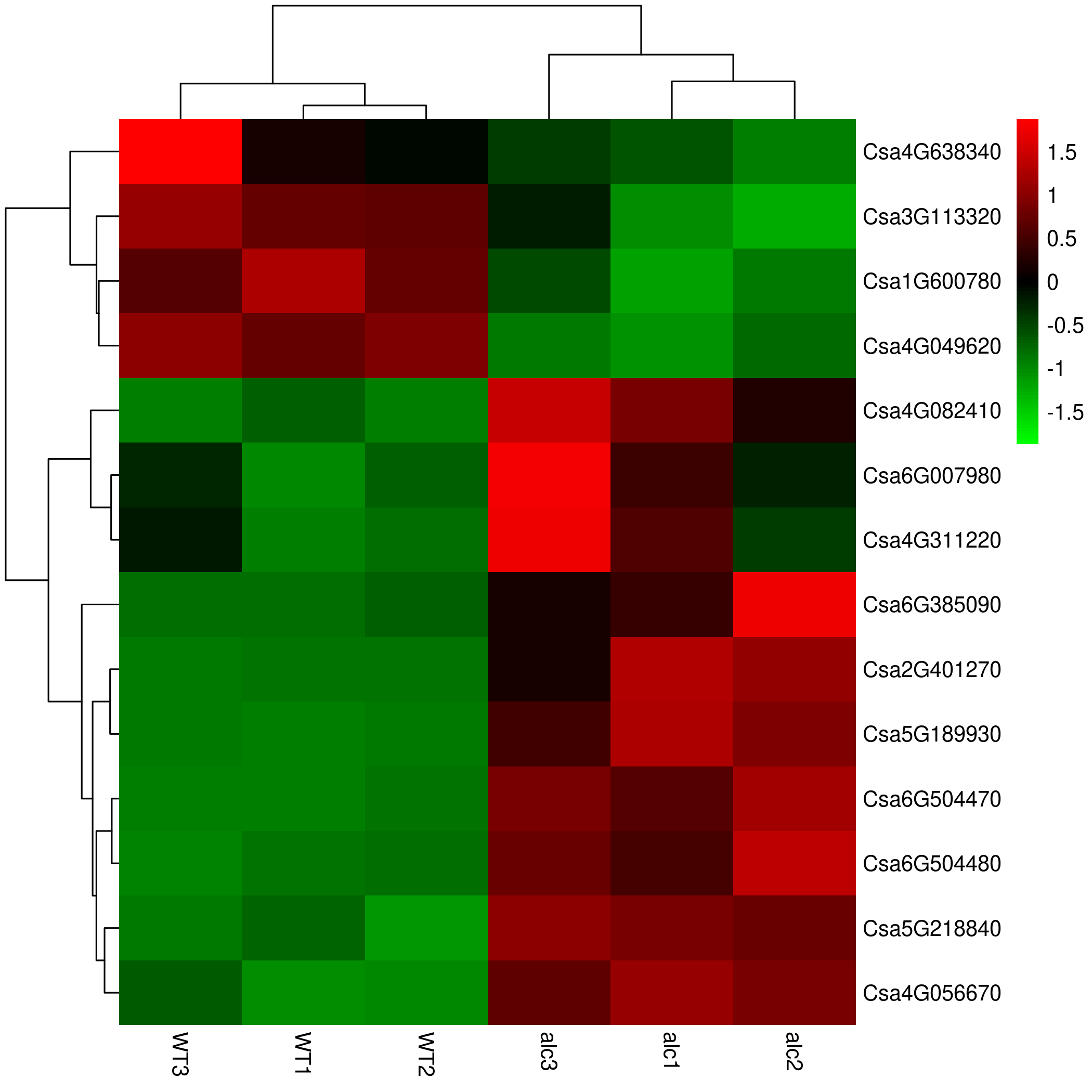

Supplement: Supplementary Figure 2 — A heat map showing expression patterns of genes related to chlorophyll metabolism, chloroplast formation and Methylerythritol 4-phosphate (MEP) pathway. [file Image_1.png]
